# Effects of High Volume Haemodiafiltration on Inflammatory Response Profile and Microcirculation in Patients with Septic Shock

**DOI:** 10.1155/2015/125615

**Published:** 2015-04-29

**Authors:** Kadri Tamme, Liivi Maddison, Rein Kruusat, Hans-Erik Ehrlich, Mirjam Viirelaid, Hartmut Kern, Joel Starkopf

**Affiliations:** ^1^Clinic of Anaesthesiology and Intensive Care, Tartu University Hospital, L. Puusepa 8, 51014 Tartu, Estonia; ^2^Department of Anaesthesiology and Intensive Care, University of Tartu, L. Puusepa 8, 51014 Tartu, Estonia; ^3^Department of Anaesthesiology and Intensive Care, DRK Kliniken, Berlin, Salvador-Allende Strasse 2-8, Köpenick, 12559 Berlin, Germany

## Abstract

*Background*. High volumes of haemofiltration are used in septic patients to control systemic inflammation and improve patient outcomes. We aimed to clarify if extended intermittent high volume online haemodiafiltration (HVHDF) influences patient haemodynamics and cytokines profile and/or has effect upon sublingual microcirculation in critically ill septic shock patients. *Methods*. Main haemodynamic and clinical variables and concentrations of cytokines were evaluated before and after HVHDF in 19 patients with septic shock requiring renal replacement therapy due to acute kidney injury. Sublingual microcirculation was assessed in 9 patients. *Results*. The mean (SD) time of HVHDF was 9.4 (1.8) hours. The median convective volume was 123 mL/kg/h. The mean (SD) dose of norepinephrine required to maintain mean arterial pressure at the target range of 70–80 mmHg decreased from 0.40 (0.43) *μ*g/kg/min to 0.28 (0.33) *μ*g/kg/min (*p* = 0.009). No significant changes in the measured cytokines or microcirculatory parameters were observed before and after HVHDF. *Conclusions*. The single-centre study suggests that extended HVHDF results in decrease of norepinephrine requirement in patients with septic shock. Haemodynamic improvement was not associated with decrease in circulating cytokine levels, and sublingual microcirculation was well preserved.

## 1. Introduction

Sepsis, defined as systemic inflammatory response syndrome (SIRS), associated with infection and acute organ dysfunction, hypoperfusion, or hypotension [1] remains a major healthcare problem [2]. While treatment of infection with antibiotics is well established [3], control of systemic inflammation is equally important but difficult to achieve due to the extremely complicated nature of the reaction and numerous mediators involved. Haemofiltration has been suggested as beneficial in restoring immunohomeostasis [4, 5]. Since the study of Ronco et al. [6], demonstrating better intensive care patient survival with increased ultrafiltration rates of renal replacement therapy (RRT), high filtration volumes have been used in septic patients with the aim of controlling systemic inflammation and improving patient outcomes. In several studies higher filtration volumes have been shown to achieve haemodynamic improvement [7–9] and possibly survival benefit [10, 11] in patients with septic shock. Although a recent randomised controlled study [12] showed no beneficial effects, the concept of attenuating the overwhelming systemic reaction to infection by nonspecific removal of a broad spectrum of pro- and anti-inflammatory mediators remains attractive. Extended daily high volume haemodiafiltration (HVHDF) is a hybrid method of intermittent RRT, where high filtration volumes are applied for 10 to 20 hours daily. The method has been shown to be a safe and cost-effective alternative for continuous venovenous haemodiafiltration (CVVHDF) [13]. In the present observational pilot study we aimed to clarify if this method of RRT influences cytokines profile and/or has effect upon sublingual microcirculation in critically ill septic shock patients.

## 2. Patients and Methods

The prospective single-centre observational study was conducted in the general intensive care unit of Tartu University Hospital from September 1, 2011, till March 1, 2014. The study was approved by the Research Ethics Committee of the University of Tartu. Informed consent from next of kin was obtained for all patients prior to study inclusion. The patient's informed consent was obtained retrospectively, if he/she recovered sufficiently.

### 2.1. Patients

Adult patients were eligible for the study, if they had severe sepsis or septic shock as defined by the ACCP/SCC Consensus Conference [1], had acute kidney injury (AKI) deemed by the treating clinician to require renal replacement therapy, based on the presence of at least one of the following criteria: (1) oliguria (urine output < 100 mL in a 6-hour period), unresponsive to fluid therapy, (2) serum potassium concentration exceeding 6.5 mmol/L, (3) severe acidemia (pH < 7.2), (4) plasma urea nitrogen level above 25 mmol/L, (5) serum creatinine concentration above 300 *μ*mol/L, or (6) presence of clinically significant organ edema (e.g., pulmonary oedema), and had an arterial line* in situ*. Patients with pregnancy and life expectancy of less than 8 hours were excluded.

### 2.2. Extended High Volume Haemodiafiltration

Extended high volume venovenous haemodiafiltration (HVHDF) in the ultracontrol predilution mode with AK 200 ULTRA S (Gambro, Lund, Sweden) as described by Kron et al. [13] was applied as clinically indicated. This mode titrates filtration volume to the maximal possible extent or volume by gradually increasing transmembrane pressure until maximal filtration rate is achieved and maintaining this pressure at maximum effectiveness. HVHDF was performed with capillary dialyzer Polyflux 210H (Gambro Dialysatoren, Hechingen, Germany) with surface area 2.1 m^2^ and ultrafiltration coefficient 85 mL/h/mmHg. Blood flow rate was kept at 200 mL/min and fluid flow rate at 500–650 mL/min. The substitution fluid was delivered prefilter. In this method the substitution fluid is taken from the dialysis fluid and thus reduces the dialysis fluid flow rate. The prescribed duration of HVHDF was 10 hours. The extent of fluid removal from the patient was prescribed by the treating clinician.

### 2.3. Biochemical Markers

Blood samples were taken from a preexisting arterial cannula immediately before the start and after the end of HVHDF session in the study and immediately centrifuged and serum was stored at −80°C until analysed.

The cytokines and growth factors were measured in sera with the Evidence Investigator Cytokine and Growth Factors High-Sensitivity Array (CTK HS Cat. number EV 3623; RANDOX Laboratories Ltd., Crumlin, UK) according to the manufacturer's protocol. Assay sensitivity varied from 0.12 pg/L to 2.12 pg/L depending on specific marker analyte. The reproducibility of the assay for individual cytokine was determined using the quality controls provided with the kit.

### 2.4. Videomicroscopy

Sublingual microcirculation was assessed in 10 patients before and after HVHDF by an SDF imaging device (Microscan; Microvision Medical, Amsterdam, Netherlands). In total, at least nine videos were taken at each time point, of which five best were analysed.

Microcirculatory videos of all patients were collected and thereafter analysed with the aid of specialized software (Automated Vascular Analysis 3.02; Academic Medical Centre, University of Amsterdam, Netherlands) by two separate investigators unaware of the study protocol.

The microcirculation cut-off value for the vessels was 20 *μ*m.

The following 2 parameters were calculated automatically by the software: (1) total vascular density (TVD) was total vessel length of the small vessels divided by the total area of the image and (2) DeBacker score was the number of small vessels crossing horizontal and vertical lines, drawn on the screen, divided by the total length of the lines [14]. Subsequent parameters were derived from the subjective analysis of blood flow in the microcirculatory videos, while perfusion was evaluated as continuous (continuous flow for at least 15 seconds), sluggish (slow but continuous flow), intermittent (no flow ≤ 50% of time), or absent (no flow ≥ 50% of time) [14]; (3) the proportion of perfused vessels (PPV) was calculated as 100 × [total number of vessels − (no flow + intermittent flow)]/total number of vessels, (4) perfused vessel density (PVD) was calculated by multiplying the vessel density by the proportion of the perfused vessels, and (5) microvascular flow index (MFI) was calculated as the mean of blood flow in four separate quadrants, while the blood flow was characterized as absent 0, intermittent 1, sluggish 2, or normal 3.

### 2.5. Statistical Analysis

All except interobserver variability calculations were performed using the Statistical Package for the Social Sciences (IBM SPSS Statistics 20.0, Somers, NY, USA) software. Normal distribution of data was confirmed by visual inspection of result histograms. Data with normal distribution are presented as mean (SD: standard deviation) and data not normally distributed as median (IQR: interquartile range). Paired* t*-test for normally distributed data and Wilcoxon matched pairs test for nonnormally distributed data were used to compare prediafiltration values against postdiafiltration values. Differences were considered significant at *p* < 0.05.

Interobserver variability was calculated separately for each parameter through the Bland-Altman analysis for assessing agreement between two opinions [15] using StatsDirect 2.7.9 software (StatsDirect Ltd., Cheshire, UK).

## 3. Results

### 3.1. Patients

Of 36 screened patients, 19 were enrolled in the study. The reasons for exclusion were absence of informed consent from next of kin (11 patients), life expectancy of <8 hours (5 patients), and age <18 years (1 patient). The demographics and clinical characteristics of the patients are shown in Table 1. Eighteen patients were mechanically ventilated and all except one received norepinephrine at the start of HVHDF. In addition 6 patients received dopamine, dobutamine, or milrinone. Bedside nurses maintained the mean arterial pressure (MAP) at the target range, defined by the treating physician (70–80 mmHg) by adjusting the dose of norepinephrine infusion. The fluid balance during the studied HVHDF was negative (from −17 mL to −2900 mL) in 15 patients and positive (from 154 mL to 884 mL) in four patients. The focus of infection was pneumonia in 6 cases, intra-abdominal infection (peritonitis, cholangitis, and infected pancreatic necrosis) in 10 cases, and infection of soft tissue in 3 cases. Sixteen patients were discharged from ICU alive; three died in the ICU: one died within 48 hours after study inclusion due to refractory septic shock and two died more than two weeks later due to multiple organ failure. The median (range) time from ICU admission to the study inclusion was 45 (6–361) hours. Seventeen patients had received renal replacement therapy before the studied HVHDF.

### 3.2. High Volume Haemodiafiltration

The studied HVHDF was performed for 10 hours in 17 patients. In 2 patients the HVHDF was terminated after 4 hours due to filter clotting. The mean (SD) time of HVHDF was 9.4 (1.8) hours. The median convective volume was 11.5 L/h (IQR 8.8–15.9) [123 mL/kg/h (IQR 100–211 mL/kg/h)]. Anticoagulation was performed with unfractionated heparin or heparin/protamine as clinically indicated.

### 3.3. Adverse Events

One patient developed atrial fibrillation during the HVHDF and one developed severe hypotension at the beginning of the procedure, stabilized with infusion therapy and temporary increase in norepinephrine dose. No other adverse events including hypothermia, hypophosphataemia, or hypokalaemia were observed.

### 3.4. Metabolic and Haemodynamic Indices

The changes in haemodynamic and metabolic parameters are shown in Table 2. The dose of norepinephrine required to maintain MAP within the target range of 70–80 mmHg decreased from 0.40 (0.43) *μ*g/kg/min to 0.28 (0.33) *μ*g/kg/min during HVHDF (*p* = 0.009). There were no statistically significant changes in heart rate, MAP, CI, body temperature, or serum lactate concentrations. Serum pH increased from 7.30 (0.1) to 7.4 (0.1); *p* = 0.013.

### 3.5. Cytokines

**Figure 1:**
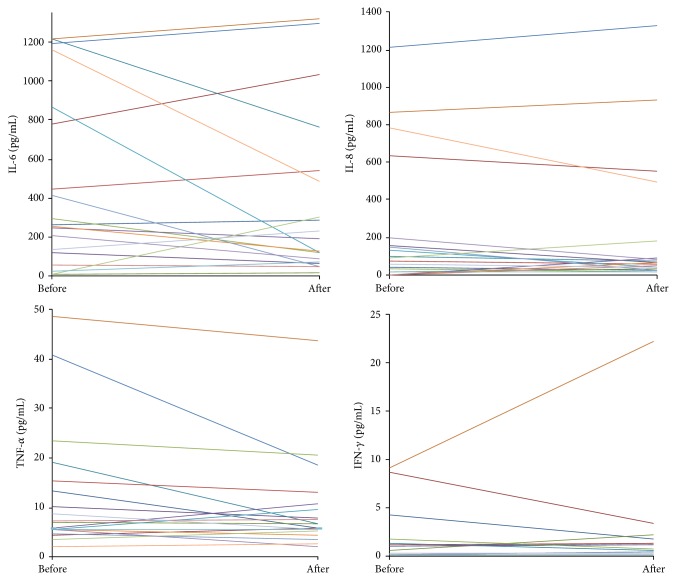
Values of main proinflammatory cytokines before and after high volume haemodiafiltration (HVHDF). The lines represent single patient values.

**Figure 2:**
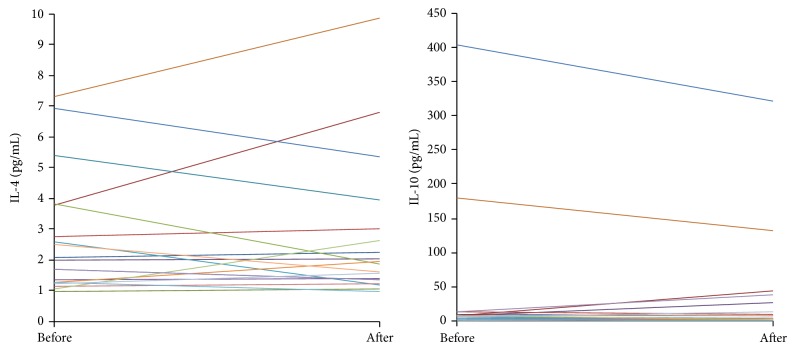
Values of main anti-inflammatory cytokines before and after high volume haemodiafiltration (HVHDF). The lines represent single patient values.

Individual changes in main proinflammatory cytokines are shown in Figure 1, and anti-inflammatory cytokines are shown in Figure 2. While high individual variability was noted, no significant differences before and after HVHDF were observed in the measured cytokines (data of IL-1*α*, IL-1*β*, and IL-2 not shown). The mean values of cytokines were significantly higher than those of healthy volunteers [16], indicating the presence of systemic inflammatory response in our patients. The ratios of pro- and anti-inflammatory mediators (IL-10/IL-6 and IL-10/TNF*α*) were not influenced by HVHDF.

### 3.6. Sublingual Microcirculation

The videos of sublingual microcirculation were analyzable in nine patients. In one patient the quality was poor because of pressure artefacts and overlighting. No significant effects were observed on measured microcirculatory parameters (Table 3).

## 4. Discussion

We found significant decrease of norepinephrine dose requirement during extended high volume (median 123 mL/kg/h) intermittent haemodiafiltration in patients with septic shock despite negative fluid balance in most patients. This finding is in line with other experimental [17] and clinical studies with different modalities of high volume haemofiltration [7–11, 18]. However, a recent randomized multicentre study, comparing continuous haemofiltration in the dose of 35 mL/kg/h to 70 mL/kg/h [12], did not show any haemodynamic or survival benefit from higher filtration volumes, yet the study was underpowered, recruiting 140 patients instead of 460 required from power calculation.

The mechanism of the effect of HVHDF on haemodynamics still remains unclear. While removal of cytokines, except TNF-*α*, occurs during HDF and is dependent on ultrafiltration rate [19], significant reduction of cytokines did not occur in our study. Besides ultrafiltration rate, substance clearance is also dependent on whether the substitution fluid is administered before or after the filter. While predilution enables achieving higher filtration volumes, it dilutes the blood before filter passage and may reduce efficiency [6]. This might be the reason we did not find significant decrease in cytokine concentrations, yet the haemodynamic effect was present.

Several hypotheses have been proposed to explain the favourable haemodynamic effects of HVHDF. The “peak concentration hypothesis” suggests that haemofiltration, applied in the early phase of sepsis, eliminates the peaks of both anti- and proinflammatory cytokines, thus restoring immunohomeostasis [4]. The hypothesis stresses the importance of applying haemofiltration early in the course of the disease. The median time from ICU admission to recruitment in our study was 45 hours, yet some patients were recruited as late as 15 days after ICU admission. This might partly account for the very different basal cytokine concentrations and their different behaviour during HVHDF. Another hypothesis, the “active transportation between two asymmetric compartments” [20] combining the “threshold immunomodulation hypothesis” [21] and the “mediator delivery hypothesis” [22], postulates that removal of cytokines from blood leads to increased gradient and therefore increased removal from tissues, thus limiting the systemic inflammation at tissue level. In addition to passive transportation, HVHF induces increase in lymphatic flow due to high amounts of crystalloids used as replacement fluids, which leads to significant drag and displacement of the cytokines to blood compartment, making them available for extracorporeal removal. This hypothesis explains why numerous studies, including ours, failed to show significant decrease in cytokine plasma concentrations, as cytokines from tissues replace those, removed from blood compartment. The “cytokinic” theory suggests that removing inflammatory mediators from plasma increases concentration gradient from plasma to infected tissues, resulting in leucocyte homing to the nidus of infection instead of passing into the blood, thus increasing bacterial clearance locally and limiting remote organ damage [23, 24].

Other mechanisms potentially responsible for the decrease in norepinephrine requirements, induced by HVHF, are cooling, which was not the case in our study, removal of unmeasured organic anions [25], or removal of mediators, responsible for hypotension and vasodilation, like prostaglandins, leukotrienes [26], and complement factors [7]. The observed increase in pH might be responsible for the decrease of vasopressor requirement in our study. Yet this explanation is unlikely, as Bellomo et al. comparing different intensities of continuous haemofiltration (CHF) found similar correction of acidosis but greater increase in mean arterial pressure and decrease in norepinephrine requirements in high intensity CHF group [25].

We did not find any changes in sublingual microcirculatory parameters. The patients were volume resuscitated before the study inclusion and had microcirculation indices close to normal [27]. While the increase on blood pressure during HVHF occurs due to increased vascular resistance rather than increased cardiac output [8], disturbances of microcirculation might be aggravated [28]. In line with other studies [29] we found no negative influence of HVHDF on microcirculation despite fluid removal during the procedure.

The present study has limitations. The small number of patients and variability in inclusion time after admission might have prevented us from detecting changes in cytokine levels. We also did not recruit a control group as this was an observational pilot study. Further, technical limitations of sublingual videomicroscopy have to be recognized. Despite these limitations, the study supports the previous suggestions that HVHDF is a safe [13, 30] and well-tolerated procedure, when vigilant monitoring and correction of serum electrolytes, especially potassium and phosphate, and warming are applied.

## 5. Conclusions

The single-centre study suggests that extended intermittent high volume haemodiafiltration results in decrease of norepinephrine requirement in patients with septic shock. Haemodynamic improvement was not associated with decrease in circulating cytokine levels, and sublingual microcirculation was well preserved. Further research is warranted to confirm these findings.

## Figures and Tables

**Table 1:** Demographics and clinical characteristics of study patients.

	Median (IQR)
Age (years)	65 (56–72)
Gender (male/female)	12/7
Weight (kg)	80 (70–100)
APACHE II	19 (16–20)
SOFA	10 (7–11)
C-reactive protein (mg/L)	252 (121–316)
Creatinine before inclusion (*μ*mol/L)	164 (127–348)
Lactate before inclusion (mmol/L)	1.5 (1.2–3.0)
ICU cumulative fluid balance before inclusion (mL)	3328 (−702–5381)
Fluid balance during HVHDF (mL)	−409 (−1405–25.75)
ICU stay (days)	23 (9–44)
ICU outcome (alive/dead)	16/3

APACHE II: Acute Physiology and Chronic Health Evaluation score II; HVHDF: high volume haemodiafiltration; ICU: intensive care unit; IQR: interquartile range.

**Table 2:** Effects of HVHDF on haemodynamic and clinical variables.

	Before HVHDF	After HVHDF	*p*
Mean arterial pressure (mmHg)	89 (71–91)	80 (73–93)	NS
Cardiac index (L/min per m^2^)	3.02 (2.5–4.2)	2.9 (2.5–3.3)	NS
Heart rate (beats/min)	102.4 (26.8)	98.5 (22.9)	NS
Body temperature (°C)	37.1 (0.6)	36.9 (0.6)	NS
Arterial pH	7.36 (0.07)	7.40 (0.06)	0.013
Norepinephrine dose (*μ*g/kg/min)	0.40 (0.43)	0.28 (0.33)	0.009
Lactate (mmol/L)	1.5 (1.2–3.0)	1.5 (1.2–2.1)	NS
SOFA	10 (7–11)	9 (7–12)	NS

HVHDF: high volume haemodiafiltration; SOFA: sequential organ failure score. Data presented as mean (standard deviation) or median (interquartile range).

**Table 3:** Sublingual microcirculatory parameters before and after HVHDF.

Parameter	Before HVHDF	After HVHDF	*p*
Total vascular density	19.4 (16.5–24.8)	21.8 (18.1–26.3)	0.139
Perfused vessel density	18.6 (15.9–22.5)	19.8 (17.7–22.9)	0.173
Proportion of perfused vessels	93.0 (88.8–95.7)	94.4 (90.3–97.1)	0.515
Microvascular flow index	2.8 (2.7–3.0)	2.9 (2.8–3.0)	0.074
DeBacker score	11.4 (9.9–14.7)	13.2 (11.1–15.2)	0.214

HVHDF: high volume haemodiafiltration. Data presented as median and interquartile range.

## References

[1] Bone R. C., Balk R. A., Cerra F. B. (1992). Definitions for sepsis and organ failure and guidelines for the use of innovative therapies in sepsis. *Chest*.

[2] Angus D. C., van der Poll T. (2013). Severe sepsis and septic shock. *The New England Journal of Medicine*.

[3] Dellinger R. P., Levy M. M., Rhodes A. (2013). Surviving sepsis campaign: international guidelines for management of severe sepsis and septic shock: 2012. *Critical Care Medicine*.

[4] Ronco C., Bonello M., Bordoni V. (2004). Extracorporeal therapies in non-renal disease: treatment of sepsis and the peak concentration hypothesis. *Blood Purification*.

[5] Servillo G., Vargas M., Pastore A. (2013). Immunomodulatory effect of continuous venovenous hemofiltration during sepsis: preliminary data. *BioMed Research International*.

[6] Ronco C., Bellomo R., Homel P. (2000). Effects of different doses in continuous veno-venous haemofiltration on outcomes of acute renal failure: a prospective randomised trial. *The Lancet*.

[7] Cole L., Bellomo R., Journois D., Davenport P., Baldwin I., Tipping P. (2001). High-volume haemofiltration in human septic shock. *Intensive Care Medicine*.

[8] Cornejo R., Downey P., Castro R. (2006). High-volume hemofiltration as salvage therapy in severe hyperdynamic septic shock. *Intensive Care Medicine*.

[9] Boussekey N., Chiche A., Faure K. (2008). A pilot randomized study comparing high and low volume hemofiltration on vasopressor use in septic shock. *Intensive Care Medicine*.

[10] Honore P. M., Jamez J., Wauthier M. (2000). Prospective evaluation of short-term, high-volume isovolemic hemofiltration on the hemodynamic course and outcome in patients with intractable circulatory failure resulting from septic shock. *Critical Care Medicine*.

[11] Joannes-Boyau O., Rapaport S., Bazin R., Fleureau C., Janvier G. (2004). Impact of high volume hemofiltration on hemodynamic disturbance and outcome during septic shock. *ASAIO Journal*.

[12] Joannes-Boyau O., Honoré P. M., Perez P. (2013). High-volume versus standard-volume haemofiltration for septic shock patients with acute kidney injury (IVOIRE study): a multicentre randomized controlled trial. *Intensive Care Medicine*.

[13] Kron J., Kron S., Wenkel R. (2012). Extended daily on-line high-volume haemodiafiltration in septic multiple organ failure: a well-tolerated and feasible procedure. *Nephrology Dialysis Transplantation*.

[14] de Backer D., Hollenberg S., Boerma C. (2007). How to evaluate the microcirculation? report of a round table conference. *Critical Care*.

[15] Bland J. M., Altman D. G. (1986). Statistical methods for assessing agreement between two methods of clinical measurement. *The Lancet*.

[16] Karu I., Starkopf J., Zilmer K., Zilmer M. (2013). Growth factors serum levels in coronary artery disease patients scheduled for bypass surgery: perioperative dynamics and comparisons with healthy volunteers. *BioMed Research International*.

[17] Bellomo R., Kellum J. A., Gandhi C. R., Pinsky M. R., Ondulik B. (2000). The effect of intensive plasma water exchange by hemofiltration on hemodynamics and soluble mediators in canine endotoxemia. *American Journal of Respiratory and Critical Care Medicine*.

[18] Chu L.-P., Zhou J.-J., Yu Y.-F., Huang Y., Dong W.-X. (2013). Clinical effects of pulse high-volume hemofiltration on severe acute pancreatitis complicated with multiple organ dysfunction syndrome. *Therapeutic Apheresis and Dialysis*.

[19] Atan R., Crosbie D., Bellomo R. (2013). Techniques of extracorporeal cytokine removal: a systematic review of the literature on animal experimental studies. *International Journal of Artificial Organs*.

[20] Honoré P. M., Jacobs R., Boer W. (2012). New insights regarding rationale, therapeutic target and dose of hemofiltration and hybrid therapies in septic acute kidney injury. *Blood Purification*.

[21] Honoré P. M., Matson J. R. (2004). Extracorporeal removal for sepsis: acting at the tissue level—the beginning of a new era for this treatment modality in septic shock. *Critical Care Medicine*.

[22] Di Carlo J. V., Alexander S. R. (2005). Hemofiltration for cytokine-driven illnesses: the mediator delivery hypothesis. *International Journal of Artificial Organs*.

[23] Namas R. A., Namas R., Lagoa C. (2012). Hemoadsorption reprograms inflammation in experimental gram-negative septic peritonitis: insights from *in vivo* and *in silico* studies. *Molecular Medicine*.

[24] Honore P. M., Jacobs R., Joannes-Boyau O. (2012). Moving from a cytotoxic to a cytokinic approach in the blood purification labyrinth: have we finally found Ariadne's thread?. *Molecular Medicine*.

[25] Bellomo R., Lipcsey M., Calzavacca P. (2013). Early acid-base and blood pressure effects of continuous renal replacement therapy intensity in patients with metabolic acidosis. *Intensive Care Medicine*.

[26] Yokoyama K., Takabayashi S., Komada T. (2009). Removal of prostaglandin E_2_ and increased intraoperative blood pressure during modified ultrafiltration in pediatric cardiac surgery. *Journal of Thoracic and Cardiovascular Surgery*.

[27] Maddison L., Riigor K. M., Karjagin J., Starkopf J. (2014). Sublingual microcirculatory changes during transient intra-abdominal hypertension—a prospective observational study in laparoscopic surgery patients. *Clinical Hemorheology and Microcirculation*.

[28] Sykora R., Chvojka J., Krouzecky A. (2009). High versus standard-volume haemofiltration in hyperdynamic porcine peritonitis: effects beyond haemodynamics?. *Intensive Care Medicine*.

[29] Ruiz C., Hernandez G., Godoy C., Downey P., Andresen M., Bruhn A. (2010). Sublingual microcirculatory changes during high-volume hemofiltration in hyperdynamic septic shock patients. *Critical Care*.

[30] Borthwick E. M. J., Hill C. J., Rabindranath K. S., Maxwell A. P., McAuley D. F., Blackwood B. (2013). High-volume haemofiltration for sepsis. *Cochrane Database of Systematic Reviews*.

